# Deciphering intratumor heterogeneity and temporal acquisition of driver events to refine precision medicine

**DOI:** 10.1186/s13059-014-0453-8

**Published:** 2014-08-27

**Authors:** Crispin Hiley, Elza C de Bruin, Nicholas McGranahan, Charles Swanton

**Affiliations:** Cancer Research UK London Research Institute, Lincoln’s Inn Fields, London, WC2A 3LY UK; Institute of Cancer Research, Old Brompton Road, London, SW7 3RP UK; University College London Cancer Institute, Huntley Street, London, WC1E 6BT UK; Centre for Mathematics & Physics in the Life Science & Experimental Biology (CoMPLEX), University College London, Gower Street, London, WC1E 6BT UK

## Abstract

The presence of multiple subclones within tumors mandates understanding of longitudinal and spatial subclonal dynamics. Resolving the spatial and temporal heterogeneity of subclones with cancer driver events may offer insight into therapy response, tumor evolutionary histories and clinical trial design.

## Tumor heterogeneity

The identification of somatic events and mutational processes that drive a cancer is increasingly important for precision cancer diagnosis and therapy. To date, sequencing efforts have identified several hundred cancer-driver mutations and genomic aberrations across multiple cancer types [1–4]. Sequencing studies have also shed light on the extent of tumor diversity, not only among tumors from different patients (intertumor heterogeneity) but also within individual tumors (intratumor heterogeneity). Pathologists have long recognized heterogeneity within tumors at the morphological level, and heterogeneity at the genetic level was first shown several decades ago by cytogenetic analyses (as reviewed by Navin and Hicks [5]), but more recent sequencing studies have provided deeper insights into the full extent of intertumor and intratumor heterogeneity. It is increasingly recognized that tumors consist of multiple genetically distinct subclones that often evolve following a pattern of branched evolution.

There is a need, therefore, not only to determine which driver events occur in a tumor but also to understand their relative timing during tumor evolution. Moreover, our understanding of how changes in the prevalence of different subclones over time impact upon therapeutic response and clinical outcome remains limited. There is an increased realization of the need to understand a tumor’s evolutionary history using both spatial and longitudinal genomic information and to identify driver events and mutational processes that contribute to tumor initiation, maintenance, progression and subclonal diversification. Here, we summarize recent findings on the relevance of subclonal driver events. We also describe how subclonal diversity might contribute to the limitations of targeted therapies and how it can be leveraged to study the evolutionary history of a tumor and to optimize clinical trial design.

## Intratumor heterogeneity and cancer evolution

**Figure 1:**
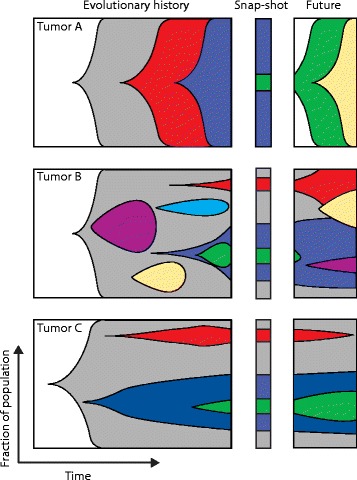
**Evolution of three tumors.** The left panel shows the evolutionary history of each tumor, the middle panel represents a snapshot of the tumor at a given time, and the right panel shows the potential future development. Tumor A shows a linear evolution pattern; tumors B and C display a branched pattern. Single snapshots of Tumors B and C may suggest that they have identical evolutionary processes, but their past and future evolution actually follow different patterns.

Subclonal populations of tumor cells arise from either random genetic drift or from the selection of cells that have a phenotypic advantage within a particular environment (for in-depth reviews on the causes of heterogeneity, the impact of genetic drift and modes of evolution, see [6–9]). In brief, tumor evolution can follow either a branched or a linear pattern, both of which can result in intratumor heterogeneity (Figure 1). A linear evolutionary pattern, whereby successive acquisition of advantageous mutations results in fitter clones that outgrow ancestral clones, results in a relatively homogeneous tumor. Some heterogeneity can result from linear evolution if a new clone has not yet fully outcompeted its predecessor. Cases of linear evolution have been observed in multiple myeloma (MM) [10] and acute myeloid leukemia (AML) [11]. A branched pattern of evolution, in which multiple distinct subclones co-exist and evolve simultaneously within a tumor, will result in a heterogeneous tumor in which there is potential for multiple subclonal driver events. Branched tumor evolution has been found in many tumor types, including breast [12], ovarian [13], prostate [14], pancreatic [15,16], and bladder cancers [17], as well as in chronic lymphocytic leukemia (CLL) [18], MM [10,19], AML [20], glioma [21] and clear cell renal cell carcinoma (ccRCC) [22,23].

The subclonal diversity within a tumor if viewed as a snapshot, rather than longitudinally, provides little information about the future evolutionary routes that subclonal populations might take. Has the dominant subclone within a tumor outcompeted less fit minor subclones or is a new fitter subclonal population emerging (Figure 1)? A greater understanding of the evolutionary timings and ‘life histories’ of tumors might shed light on the most clinically significant subclones and reveal common rules that govern tumor evolution both within and across cancer subtypes.

## Intratumor heterogeneity illuminates a tumor’s life history

**Figure 2:**
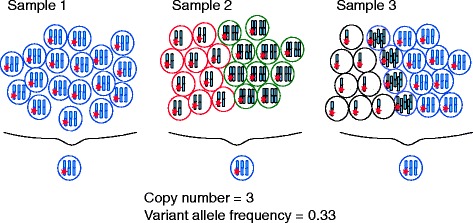
**Somatic aberrations in cancer cell populations.** The DNA copy number and number of mutant alleles (red stars) within single cancer cells can be difficult to discern when looking at a whole population of cancer cells. Samples 1-3 on average each have three copies of a particular chromosome, and a variant allele frequency of 0.33, but the collection of cancer cells in each population are vastly different. Single-cell sequencing may be required to elucidate the underlying population structure.

Deciphering genetic intratumor heterogeneity can reveal the temporal composition of genetic events that take place during the disease course. Bioinformatics tools such as ABSOLUTE [24] and PyClone [25] integrate data on variant allele frequency, local copy number and tumor purity, and can give estimates of the clonality of somatic events, even within individual tumor biopsies. These estimates can be refined through multi-region sequencing approaches that reveal both the clonality and the spatial composition of tumor subclones, showing that mutations can be clonal in one tumor region but completely absent in another tumor region [13,16,22]. Nevertheless, regions within a tumor still contain many cells, and clonality analysis will be unable to resolve the subclonal composition of a tumor beyond the resolution of the sample taken and used for analysis. When comparing samples containing many cells, multiple permutations of the distribution of mutations, or changes in copy number, across the individual cells can result in similar variant allele frequencies and local copy numbers among samples (Figure 2). Therefore, single-cell sequencing will ultimately be required to determine unequivocally the true number of different subclones within a population and to characterize them without aggregating the results from multiple cells within a sample.

**Figure 3:**
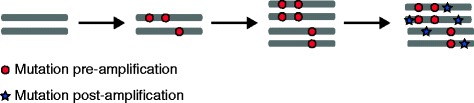
**Timing of mutations.** The number of copies of a mutation can shed light on when it occurred. A mutation that is acquired before a chromosome doubling event will be present on multiple chromosome copies, whereas a mutation acquired after the doubling event will be present on only one chromosome copy.

With regards to evolutionary timing, clonal somatic mutations that are present in all tumor cells will have been acquired relatively ‘early’ in tumorigenesis, before or during the appearance of the most-recent common ancestor. These early mutations are a mix of both driver events that contributed to tumor initiation and passenger mutations that may have preceded transformation. Conversely, subclonal mutations, which are present in only a subset of tumor cells, represent ‘later’ events occurring after the appearance of the most-recent common ancestor and so after tumor initiation. Clonal and subclonal mutations can be further temporally dissected by looking at chromosomal amplifications; mutations acquired before amplification will be present on at least two chromosome copies, whereas mutations acquired after amplification will be present on only one copy [26–28] (Figure 3).

In breast and pancreatic cancers, the majority of known mutational and copy number driver events are relatively early events [15,16,27,29]. In ccRCC, however, the majority of identified driver mutations were found to be subclonal. In fact, inactivating mutations in the Von Hippel-Lindau tumor suppressor gene and loss of heterozygosity at chromosome 3p were the only somatic events occurring ‘early’ in ten ccRCC tumors analyzed [22]. Conversely, mutations in certain driver genes, including *BAP1* (BRCA1 associated protein-1), *PTEN* (phosphatase and tensin homolog), *PIK3CA* (phosphatidylinositol-4,5-bisphosphate 3-kinase, catalytic subunit alpha), *SETD2* (SET domain containing 2) and *TP53* (encoding tumor protein p53), were always subclonal, and thus probably involved in ccRCC progression. Selection for mutations in these genes during later stages of ccRCC development is evidenced by the observation that different subclones acquire mutations in the same gene in parallel.

Driver events are not always clonally dominant across all cancer subtypes. For example, loss of the tumor suppressor PTEN has been identified, by sequencing and fluorescence *in situ* hybridization analyses, as a subclonal event in prostate tumors but often as a clonal event in triple negative breast cancer [14,29,30]. Similarly, TP53 mutations were usually identified as early events in triple negative breast tumors and cutaneous squamous cell carcinomas [28,29] but predominantly as subclonal in CLL and ccRCC [18,22]. Nevertheless, to define a clonal mutation truly as an initiating event, single cell analysis would be required to reconstruct the evolutionary lineage of a sample. For example, the etiological significance in MM of the chromosomal translocation t(11;14) was further evidenced by the fact that this was the sole abnormality in some subclones and that genetic variegation of this mutation in an initial clone had resulted in the tumor heterogeneity [31].

Deciphering genetic intratumor heterogeneity sheds light not only on the temporal acquisition of somatic driver events, but also on the temporal dynamics of mutational processes. Analysis of breast cancers found that early mutations were dominated by C-to-T transitions, predominantly in a CpG context, probably reflecting spontaneous deamination of methylated cytosines [32]. By contrast, in ccRCC, C-to-T transitions at CpG sites were enriched in late mutations [22]. Later in breast cancer evolution, novel mutational processes, such as mutagenesis by APOBEC (apolipoprotein B mRNA-editing, enzyme-catalytic, polypeptide-like 3G) cytidine deaminases, were found to dominate in certain samples [32]. Intriguingly, in MM, the contribution of APOBEC-edited mutagenesis was found to either increase or decrease over time, depending on the sample [10], whereas in bladder cancer, the contribution of APOBEC remained relatively stable between pairs of superficial noninvasive and mucosa- or muscle-invasive tumors from two patients [17].

Taken together, these studies highlight the diversity in tumor evolutionary processes, with variation in both the temporal acquisition of driver mutations and the mutational signatures themselves observed both within and between tumor types. As more samples are analyzed, it will be important to validate which driver genes are always clonal (that is, on the trunk of a tumor’s evolutionary tree) and which may occur later in tumor evolution, driving subclonal expansions. Early drivers may serve as optimal therapeutic targets in future drug-development strategies [33]. Importantly, whereas a clonal driver mutation in a tumor is fixed and will remain clonal unless subject to copy number loss later in tumor evolution, a subclonal driver mutation may become clonal, evade detection or disappear entirely at a later stage of tumor evolution. Thus, a subclonal driver is more dynamic than a clonal driver. Longitudinal studies are needed to shed light on whether there are epistatic relationships between driver events and to explore the possibility of sets of evolutionary rules that determine how tumor cell populations change over time within each cancer type. The existence of such evolutionary rules is supported by evidence of the parallel evolution of distinct subclones within the same tumor, each harboring distinct somatic events that affect the same gene or signal transduction pathway, and by pairwise associations between different driver events [22,31,34]. Conceivably, if rules regarding the temporal acquisition of somatic driver events can be defined - taking into account the tumor microenvironment, the host genome and early somatic events in tumor evolution - they could inform therapeutic strategies. Similarly, a greater understanding of whether mutational processes, such as APOBEC-mediated mutagenesis, generally occur transiently or accumulate gradually over time may shed light on how specific cancers should be monitored and treated [35].

## Clinical relevance of spatial and temporal heterogeneity

Heterogeneity between primary and metastatic lesions has profound implications for approaches to genomic research and patient care, as does the heterogeneity of clones within a single sample that changes over time because of cell-intrinsic mechanisms such as genomic instability or selective pressures from tumor-directed therapy.

Spatial heterogeneity in solid tumors can result in significant sampling bias. In high-grade serous ovarian cancer, multi-region sampling of six patients prior to treatment demonstrated the diversity of somatic mutation, copy number and gene expression within each patient [13]. Other than *TP53,* few driver genes were ubiquitous in the multiple sampled regions from each patient. In ccRCC, multiple biopsies are required to better define the true extent of genomic heterogeneity and clinically relevant mutations. For example, mutations in the mammalian target of rapamycin kinase that confer resistance to everolimus can be found in some but not all tumor regions [22,23].

Sequencing studies have demonstrated heterogeneity of driver events between primary tumor and metastatic sites. Similar heterogeneity has also been demonstrated for clinically relevant biomarkers [12,16,23]. A retrospective review of HER2 (v-erb-b2 avian erythroblastic leukemia viral oncogene homolog 2) status in primary breast cancer and metastatic relapses showed significant discordance [36]. Patients with stage IV breast cancer who received HER2-directed therapy due to having had HER2-positive early breast cancer and who on retrospective analysis were found to have a HER2-negative metastatic relapse had shorter overall survival compared to those who had a true HER2-positive metastatic relapse. Sequencing of recurrent high-grade gliomas, after earlier surgical resection and sequencing of low grade lesions, showed that almost half of the high-grade relapses did not come from the previously resected low-grade glioma but from an ancestral clone that predated the low-grade component [21]. Many potentially therapeutically targetable driver mutations, such as BRAF (B-Raf proto-oncogene, serine/threonine kinase) V600E, that were present in the primary low-grade lesion, were not present in the recurrent high-grade relapse. Multi-region sequencing and expression analysis from glioblastoma multiforme (GBM) patients have also shown that heterogeneity results in the presence of multiple tumor subtypes, as identified by gene expression classifiers, within the same tumor [37,38]. This questions the utility of such classification systems and gene expression signatures to define individual GBM subtypes.

The sequencing of two temporally separated CLL samples from treated and untreated patients showed that many of these tumors underwent clonal evolution and that the presence of a subclonal driver was an independent risk factor for disease progression [18]. Conversely, in myelodysplastic syndrome, driver mutations had a similar prognostic significance whether they were clonal or subclonal; the absolute number of driver mutations rather than clonality had the biggest implications for outcome [34]. The impact of intratumor heterogeneity and the clonality of driver mutations on prognosis and response to precision medicine has not been studied prospectively. A UK-based longitudinal observational study, Tracking Non-small Cell Lung Cancer Evolution Through Therapy (TRACERx), has been launched to assess this [39]; 842 patients will have whole-exome sequencing (WES) of multiple regions of their resected primary tumor, as well as of cell-free DNA (cfDNA) and circulating tumor cells (CTCs) at multiple time points throughout follow-up. Patients who suffer disease recurrence will be consented for repeat tumor sampling in order to define the evolutionary routes of individual tumors. This national study may provide insight into the role of intratumor heterogeneity and the (sub)clonality of driver events on outcome, reveal the origins of the lethal subclone and begin to define selection pressures initiated by therapy.

## Clonal evolution, heterogeneity and cancer therapy

A number of studies have demonstrated that treatment can act as a selection pressure in some malignancies, driving clonal evolution and selecting for certain subclones. This emphasizes the importance of longitudinal tumor sampling strategies to depict tumor genomic landscapes. In acute lymphoblastic leukemia, copy number abnormalities (CNA) were strikingly different between samples taken at diagnosis and after relapse following chemotherapy [40]. On retrospective analysis, the cells responsible for relapse were present as a subclonal population at diagnosis; chemotherapy had selected for a population with CNA in genes involved in the regulation of the cell cycle and B-cell development. In CLL, cancers were more likely to have undergone clonal evolution in patients treated with chemotherapy than in untreated patients [18]. In non-small cell lung cancer (NSCLC), patients who relapsed with MET-amplified epidermal growth factor receptor (EGFR) tyrosine kinase inhibitor (TKI)-resistant disease following treatment with an EGFR inhibitor harbored a low-frequency subclone (<1% of cells) with *MET* amplification prior to treatment, which was selected for during therapy [41]. Notably, a subclonal population with *MET* amplification at such a low frequency would be difficult to identify in a heterogeneous biopsy sample. Conceivably, these patients may have benefited from combination EGFR TKI and MET inhibition to forestall selection of the drug-resistant subclone.

Similarly, the presence of the EGFR T790M mutation, which is associated with resistance to EGFR TKI therapy, has been demonstrated prior to treatment with EGFR inhibition in patients with NSCLC [42]. In this study, matrix-assisted laser desorption/ionization mass spectrometry and next-generation sequencing (NGS) were used to detect the presence of low-frequency (<5% of cells) T790M mutations in pre-treatment samples that were not detected using standard Sanger sequencing. They found that a greater prevalence of the T790M subclonal population was detected in post-treatment biopsies and, in the context of selection due to EGFR TKI, the T790M mutation acted as a driver of subclonal expansion.

In a study of patients with colorectal cancer, multiple somatic mutations in KRAS (Kirsten rat sarcoma viral oncogene homolog), which are associated with resistance to anti-EGFR antibody therapy, could be detected non-invasively through cfDNA analysis during the acquisition of drug resistance, and were predicted to be present in a subclone prior to treatment [43]. By contrast, a recent sequencing study of five patients with RAF/MEK-inhibitor-resistant BRAF melanoma found no evidence of a pre-existing resistant subclonal population, suggesting that resistant tumor cells had developed *de novo* on treatment, or that resistant subclones were present in the pre-treatment tumor at frequencies that were below the limits of detection [44].

Cancer therapies not only can act as the selection pressure to drive tumor evolution along a certain lineage if pre-existing subclones possess genotypes that are associated with a drug-resistant phenotype [18,19] but also can generate new subclonal driver events. For example, temozolamide, the standard first-line therapy for GBM, induces mutations in tumor DNA. Some are deleterious for the cell and result in death, others neutral and act as passenger mutations, but others such as mutations in mismatch repair (MMR) genes are potentially advantageous for tumor cells. Some GBM tumors treated with temozolamide exhibited a mutator phenotype, resulting from mutations in MMR genes, and were found to harbor driver mutations in *RB1* (encoding retinoblastoma 1), *PIK3CA* and *PTEN* that bore the signature of temozolamide-induced mutagenesis [21,45].

Greater understanding of resistance mechanisms (Table 1) suggests that more emphasis should be placed on the longitudinal analysis of tumors in the clinical setting and on the use of combination or adaptive sequential therapy to manage the selection of resistant subclones [46]. The use of combinations of precision therapies to forestall resistance will result in a greater burden of toxicity for patients and has implications for health economics. It remains unclear whether such approaches will sufficiently address the presence of subclonal drivers in advanced disease. In this regard, immune-modulatory strategies seem compelling in order to adapt to the changing cancer genomic landscape.

**Table 1:** **Mechanisms of resistance to common cytotoxic chemotherapies and precision medicines**

**Systemic agent**	**Target**	**Resistance mechanism**
**Platinum-based chemotherapy**	DNA	Decreased mismatch repair proficiency (e.g.↓MLH1 & ↓MSH2)
**● cisplatin**		
**● carboplatin**		Increased efficiency of other modes of DNA repair (e.g. nucleotide excision repair – ↑ERCC1, trans lesion synthesis – ↑POLH, homologous recombination – BRCA1/2 restoration)
**● oxaliplatin**		
**Microtubule-targeting chemotherapy**	Tubulin	Drug efflux via increased expression of MDR-1
		Changes in microtubule structure (e.g. mutations in β-tubulin and overexpression of βIII-tubulin
**● docetaxel**		
**● vinorelbine**		
		Chromosomal instability
**EGFR TKi**	EGFR TK domain	Resistance mutation, e.g. T790M
**● erlotinib**		
		MET amplification
**● gefitinib**		
		EGFR amplification
		Transformation to small cell lung cancer
**EGFR monoclonal antibody**	EGFR extracellular domain	Acquired KRAS or NRAS mutation
		Activation of PIK3CA/PTEN pathway
**● cetuximab**		
**● panitumumab**		
		Inhibition of cetuximab binding, e.g. EGFR-S492R
**BRAF TKi**	BRAF-V600E	Elevated BRAF/CRAF/COT1 expression
**● vemurafenib**		
**● dabrafenib**		Acquired mutation in other elements of the MAPK pathway
		Persistent activation of other kinases, e.g. EGFR and PDGFRβ
**ALK TKi**	EML4-ALK	Secondary EML4-ALK mutations or rearrangements
**● crizotinib**		CD74-ROS1 rearrangement
**● ceritinib**		

See [46–52] for more detailed review. ALK, anaplastic lymphoma kinase; BRAF, B-Raf proto-oncogene, serine/threonine kinase; BRCA1/2, encoding breast cancer 1/2, early onset; COT1, cancer Osaka thyroid oncogene 1; CRAF, Raf-1 proto-oncogene, serine/threonine kinase; EGFR, epidermal growth factor receptor; EML4, echinoderm microtubule associated protein like 4; ERCC1, excision repair cross-complementation group 1; MAPK, mitogen-activated protein kinases; MDR1, multi-drug resistance 1; MET, MET proto-oncogene, receptor tyrosine kinase; MLH1, mutL homolog 1; MSH2, mutS homolog 2; PDGFR, platelet-derived growth factor receptor; PIK3CA, PI3K catalytic subunit α; POLH, DNA polymerase H; PTEN, phosphatase and tensin homolog; ROS1, ROS proto-oncogene 1; TKi, tyrosine kinase inhibitor.

In some cases, it is becoming clear that resistance to therapy can be mediated by more than one resistant subclone. In colorectal cancer, different mutations in KRAS (exon 12 and 13), associated with resistance to EGFR monoclonal antibody therapy, were found in the same patient [43]. In anaplastic lymphoma kinase (ALK)-positive, crizotinib-treated NSCLC patients, resistance was driven by multiple mechanisms, such as secondary ALK mutations, amplification of KIT or the EML4-ALK fusion gene itself and EGFR pathway activation, with multiple resistance mechanisms sometimes found simultaneously within the same tumor [47]. Paradoxically, precision medicines may have a detrimental effect in the presence of polyclonal disease with subclonal driver events. BRAF inhibition has been shown to have significant anti-tumor efficacy in BRAFV600E mutant cancers. In BRAF wild-type cells, however, activation of extracellular-signal-regulated kinase signaling with the use of BRAF inhibitors can promote tumorigenesis [53]. In the context of a polyclonal tumor where a BRAF-activating mutation is subclonal in nature with the presence of BRAF wild-type subclones, BRAF inhibition might promote the growth of the BRAF wild-type population, particularly if these subclones harbor KRAS or NRAS (neuroblastoma RAS viral (v-ras) oncogene homolog) driver mutations [19]. The potential emergence of polyclonal resistance and the presence of subclonal drivers should be considered when designing clinical trials to forestall resistance to targeted agents.

The extent to which the presence of subclonal drivers and intratumor heterogeneity impacts upon the different responses witnessed with both systemic chemotherapy and precision medicines remains unclear [23,54–57]. Mixed responses to drug treatments are common and there is no consensus in clinical decision-making in this context [58]. Should the treating physician switch systemic therapy, add a second systemic therapy to combat resistance, advocate locoregional approaches with surgery or radiotherapy, or monitor clinically insignificant progression and continue therapy with the caveat that non-responding lesions might act as an evolutionary sink that later contribute to more widespread progression? Further cataloguing of clonal and subclonal drivers and common mechanisms for resistance to treatment, together with adapting clinical trial design to the challenges of tumor evolution may improve patient care in the future.

## The impact of heterogeneity and tumor evolution on the use of biomarkers for patient stratification

The identification prior to therapy and subsequent management of low-frequency subclones harboring driver events that influence clinical outcome is clearly a major challenge. Such subclones may be present at a low frequency within one biopsy, could be spatially separated within a primary tumor or might be differently distributed in the primary tumor and its metastases. In recent years, clinical trial designs have moved from stratification that is based on histology to classification by molecular subtype [59,60]. The next generation of clinical trial design has seen the development of basket trials (MATCH, I-SPY, FOCUS4 and MATRIX) that contain multiple molecular subgroups, each treated with a different therapy, that are based on pre-specified somatic aberrations. These trials often have an adaptive design that allows removal of poorly performing arms during the course of the trial [61]. A potential limitation of this approach is that, in the metastatic setting, molecular biomarker analysis for patient stratification is often performed on archival material, which may not reflect the current state of disease. This strategy may readily identify clonally dominant drivers that occur earlier in tumor evolution, but its ability to detect subclonal drivers will depend on the prevalence of that subclone within the tissue taken for analysis.

**Figure 4:**
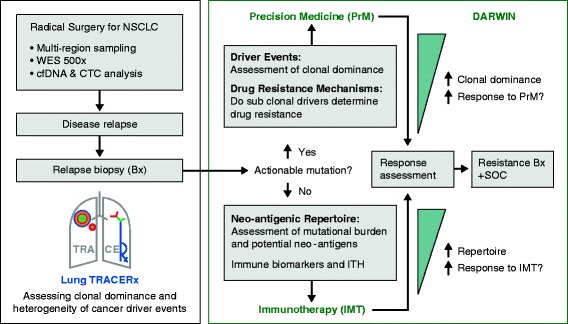
**Schematic overview of the Tracking Non-small Cell Lung Cancer Evolution Through Therapy (TRACERx) observational cohort study and how this is linked with the Deciphering Anti-tumour Response and evolution With INtratumour heterogeneity (DARWIN) trials program.** Multi-region sampling with ultra-deep 500x coverage whole-exome sequencing (WES) will be used to characterize tumor heterogeneity. Tumor heterogeneity and clonal dynamics may affect the response to precision drugs. Only patients from the TRACERx observational study will be eligible for a DARWIN trial. Therefore, in comparison to other molecularly stratified studies TRACERx & DARWIN provide a unique opportunity to study the affect of intratumour heterogeneity and clonal architecture on patient outcome. The effect of tumor heterogeneity and mutational burden on anti-tumor immunity will also be assessed through an immunotherapy arm. Bx, biopsy; CTC, circulating tumor cell; cfDNA, cell free DNA; IMT, immunotherapy; ITH, intertumor heterogeneity; NSCLC, non-small cell lung cancer; PrM, precision medicine; SOC, standard of care.

Patients from the UK-based TRACERx multi-region sequencing longitudinal observational study of NSCLC who relapse with locally advanced or metastatic disease will be eligible for the Deciphering Anti-tumor Response and evolution With INtratumour heterogeneity (DARWIN) clinical trials program (Figure 4). Patients will be allocated into molecularly stratified subgroups at the time of relapse with the *a priori* knowledge of the clonal frequency of the driver event at the time of surgery and at relapse, the latter being provided by analysis of a repeat biopsy of the metastatic site and by cfDNA and CTC analysis. These analyses will help to determine whether targeting clonally dominant drivers improves progression-free survival and how subclonal driver events impact upon disease progression and drug resistance. In the future, knowledge of dominant and subclonal drivers and resistance mechanisms may allow more optimal treatment allocation. WES will also allow assessment of the protein-coding mutational burden and the potential neo-antigenic repertoire of each tumor. This information can then be correlated to the response to immunotherapy of those without an actionable mutation for which there is an approved precision medicine.

Without the use of single-cell approaches, addressing whether subclonal populations that have resistance to therapy are present prior to treatment or are selected during therapy is hindered by the limit of detection of low-frequency cancer cell populations. Conventional Sanger sequencing can detect variants down to the level of approximately 10% prevalence. Some studies have used mutation-specific PCR strategies followed by targeted sequencing or mass spectrometry to enrich low-frequency populations within archival material, allowing detection of subclonal events at 0.1% prevalence [62,63]. Massive parallel sequencing offers the potential to identify unknown subclonal populations. Nevertheless, the optimal detection of low-frequency subclones might in some cases require multi-region analysis, or analysis of circulating tumor DNA, to identify spatially separated populations; alternatively, high sequencing depth might be used to identify low frequency but potentially clinically significant events within single samples. In addition, the error rates of NGS, resulting from PCR errors during library preparation, instrument errors during cluster amplification and image analysis, or errors from base-calling algorithms, introduce a significant false-positive rate. Using molecular barcodes to build read families from sequencing data will help to reduce the errors generated during sequencing but not PCR errors introduced during library preparation [64,65]. The challenge of detecting significant subclonal driver mutations within the statistical noise generated by high-coverage NGS will need to be addressed to allow clinical application.

## Impact of tumor heterogeneity on the use of precision medicines

The advent of NGS has permitted greater resolution in determining the extent of heterogeneity within individual tumors. Distinguishing early and late somatic events may allow a better understanding of the genes and mutational processes that are involved in tumor initiation in comparison with those involved in maintenance and metastasis, which in turn might inform new therapeutic avenues. Moreover, the distinction of clonally dominant events from subclonal driver events might allow the acceleration of drug development towards targeted early, truncal drivers of disease. Although targeting clonally dominant drivers may make intuitive sense, some trunk drivers may only be relevant for tumor initiation and targeting these drivers after clonal diversification might not be efficacious.Furthermore, the origins of the ‘lethal’ subclone or subclones might be determined by somatic events that occur later in tumor evolution and not in the dominant clone. Therefore, efforts to limit disease progression might require a greater understanding and optimal targeting of subclonal driver events.

High-throughput functional assessment of validated mutations will be important to assess the significance of these mutations and to avoid wasting resources on the further investigation of sequencing artifact or passenger mutations. The wealth and depth of data may allow us to find unanticipated gene-gene interactions and might reveal new unappreciated cancer drivers that are involved in epistatic interactions [66]. Clinical translation of these findings will be key. Novel clinical trial approaches that consider clonal evolution in the context of cancer diversity may shed light on the efficacy of unexpected combination therapies and provide evidence for adaptive therapy to avoid the selection of drug-resistant subclones [39,67–69].

## Abbreviations

ALK: Anaplastic lymphoma kinase
AML: Acute myeloid leukemia
APOBEC: Apolipoprotein B mRNA-editing, enzyme-catalytic, polypeptide-like 3G
BAP1: BRCA1 associated protein-1
BRAF: B-Raf proto-oncogene, serine/threonine kinase
ccRCC: clear cell renal cell carcinoma
cfDNA: cell-free DNA
CLL: Chronic lymphocytic leukaemia
CNA: Copy number abnormalities
CTC: Circulating tumour cell
DARWIN: Deciphering Anti-tumour response and evolution with INtratumour heterogeneity
EGFR: Epidermal growth factor receptor
GBM: Glioblastoma multiforme
HER2: v-erb-b2 avian erythroblastic leukemia viral oncogene homolog 2
KRAS: Kirsten rat sarcoma viral oncogene homolog
MM: Multiple myeloma
MMR: Mismatch repair
NGS: Next-generation sequencing
NSCLC: Non-small cell lung cancer
PCR: Polymerase chain reaction
PIK3CA: Phosphatidylinositol-4,5-bisphosphate 3-kinase, catalytic subunit alpha
PTEN: Phosphatase and tensin homolog
RB1: Encoding retinoblastoma 1
SETD2: SET domain containing 2
TKI: Tyrosine kinase inhibitor
TP53: Encoding tumor protein p53
TRACERx: Tracking non-small cell lung cancer evolution through therapy
WES: Whole-exome sequencing
